# Calcitriol ameliorates renal damage in a pre-established proteinuria model

**DOI:** 10.3892/mmr.2015.3555

**Published:** 2015-03-27

**Authors:** EDGAR MAQUIGUSSA, CARINE P ARNONI, LUCIANA G PEREIRA, MIRIAN A BOIM

**Affiliations:** Department of Medicine, Renal Division, Federal University of São Paulo, São Paulo 04339-032, Brazil

**Keywords:** vitamin D, renal fibrosis, proteinuria, angiotensin II, epithelial mesenchymal transition, transforming growth factor β

## Abstract

Proteinuria is critical in the tubulointerstitial changes that ultimately lead to renal insufficiency. Increased protein filtration has direct toxic effects on tubular epithelial cells, leading to epithelial mesenchymal transition (EMT) to a myofibroblast phenotype. Angiotensin II and transforming growth factor (TGF)-β1 are the main mediators of EMT. Calcitriol may exert a potential renoprotective effect by reducing the activity of the renin angiotensin system by suppressing renin gene expression and also by inhibiting the proinflammatory nuclear factor-κB pathway. The present study investigated the benefits of calcitriol treatment in a puromycin-induced protein-uric nephropathy model. Uninephrectomized adult male Wistar rats received intraperitoneal administration of a single dose of puromycin (100 mg/kg) or vehicle. After eight weeks, the animals were divided into two groups and received vehicle or calcitriol (0.5 *μ*g/kg) for four weeks. The vehicle-treated, proteinuric rats developed progressive proteinuria and tubulointerstitial fibrosis after 12 weeks. Increased collagen deposition and fibrosis were significantly ameliorated by calcitriol treatment. Calcitriol was effective in preventing an increase in the EMT markers, α-smooth muscle actin and fibroblast-specific protein 1, reducing macrophage infiltration as evidenced by levels of ED-1. In addition, calcitriol increased the anti-inflammatory cytokine interleukin-10 and reduced the pro-oxidant p47 phox enzyme. These effects were paralleled by a reduction in TGF-β/Smad3 expression. Calcitriol may have therapeutic potential in the proteinuric nephropathy model used in the present study by inhibiting the TGF-β1 axis.

## Introduction

Puromycin aminonucleoside (PAN) is widely used as a model of nephrotic syndrome and focal segmental glomerulosclerosis. Dysfunction in the slit diaphragm caused by PAN is associated with the development of massive proteinuria. Although glomerular injury is the primary effect of PAN, renal handling of the excess filtered proteins may contribute to tubulointerstitial lesions. The excess of protein delivered to the proximal tubule results in inflammation, tubular epithelial mesenchymal transition (EMT) and interstitial fibrosis (1,2).

Previous studies have demonstrated a significant renoprotective effect of the active form of vitamin D, or calcitriol (1,25-dihydroxycholecalciferol), in kidney diseases of various etiologies (3). Calcitriol activity is mediated through the vitamin D receptor (VDR), a member of the nuclear receptor superfamily (4). Administration of paricalcitol, a vitamin D analogue, reduced glomerulosclerosis and proteinuria, which prevented podocyte injury in a model of adriamycin-induced nephropathy (5). In addition, paricalcitol protected the kidneys against renal damage in obstructive nephropathy (6), possibly due to its ability to preserve tubular epithelial integrity via EMT prevention. Calcitriol regulates two major pathways involved in a number of pathological processes, the renin angiotensin system (RAS) (7) and the nuclear factor (NF)-κB pathway (8). Calcitriol has been well established as a negative regulator of the RAS by suppressing the prorenin gene (9). In addition, NF-κB, a major mediator of the immune response, is involved in regulating inflammatory cytokines and chemo-kines, including monocyte chemotactic protein 1, plasminogen activator inhibitor-1 and tumor-necrosis factor (TNF)-α, which are important in renal damage by inducing inflammation and fibrogenesis (10). Calcitriol may interfere with NF-κB signaling by inducing the formation of a complex between the VDR and the p65 subunit of NF-κB, preventing the complex from binding to DNA (8).

In the present study, the effect of calcitriol in PAN-induced nephrotic syndrome in rats was investigated. It was also determined whether calcitriol is beneficial for minimizing renal damage in a pre-established model of proteinuria.

## Materials and methods

The experimental protocol was approved by the Ethical Committee of the Federal University of São Paulo (CEP 0741, UNIFESP, São Paulo, Brazil). The study used 12-week-old male Wistar rats (150–200 g) supplied by the animal facility of the Federal University of São Paulo. The rats were housed in cages with *ad libitum* access to standard rat chow and tap water, in a temperature-controlled environment (23°C) with a 12 h light/dark cycle. One week prior to PAN administration, the right kidney was removed under anesthesia with 40 mg/kg ketamine and 20 mg/kg xylazine (Syntec, Hortolândia, Brazil). Nephrosis was induced using a single intraperitoneal injection of PAN (100 mg/kg body weight; Sigma-Aldrich, St. Louis, MO, USA). The animals were divided into three groups: Control (CTL; n=5), PAN treatment (PAN; n=5) and PAN combined with calcitriol treatment (PAN + calcitriol; n,5). Calcitriol (Abbott, Milan, Italy) treatment started eight weeks after PAN administration when proteinuria was established. Calcitriol was administered by subcutaneous injection (0.5 mg/kg bodyweight) five times a week for four weeks. All animals were sacrificed 12 weeks after the onset of PAN administration.

Periodically, retro-orbital blood samples were obtained from the animals under ketamine and xylazine anesthesia. Additionally, 24-h urine samples were collected in metabolic cages (Tecniplast, Buguggiate, Italy). A colorimetric assay was used to measure concentrations of creatinine (Creatinine kit; Labtest Diagnóstica, Lagoa Santa, Brazil), calcium (Arsenazo III kit; Labtest Diagnóstica) and inorganic phosphate (Inorganic Phosphorous kit; Beckman Coulter, Miami, FL, USA). Urine protein was measured using a colo-rimetric assay (Sensiprot; Labtest Diagnóstica). At completion of the experimental protocol, the animals were anesthetized with ketamine and xylazine, blood was collected from the abdominal aorta and the remaining kidney was excised. Animals were sacrificed via anesthetic overdose (160 mg/kg ketamine and 80 mg/kg xylazine; Syntec). For the mRNA and protein expression analyses, the kidney samples were immediately frozen in liquid nitrogen and kept at −80°C until use. For the histochemical and immunohistochemical analyses, the kidney samples were fixed in tamponated formaldehyde (Merck KGaA, Darmstadt, Germany) and following several washes in ethanol (Merck KGaA) and xylene (Labsynth, Diadema, Brazil), the samples were embedded in paraffin wax (Labsynth).

### Reverse transcription quantitative polymerase chain reaction (RT-qPCR)

Total RNA was purified from the whole kidney using the phenol and guanidine isothiocyanate-cesium chloride method with TRIzol^®^ (Gibco-BRL, Gaithersburg, MD, USA), according to the manufacturer’s instructions. Total RNA (2 *μ*g) was treated with DNase (RQ1 RNase-free DNase; Promega, Madison, WI, USA) to avoid genomic DNA contamination and reverse-transcribed into cDNA by adding a mixture containing 0.5 mg/ml of oligo(dT) (Invitrogen Life Technologies, Carlsbad, CA, USA), 10 mM DL-dithiothreitol (Invitrogen Life Technologies), 0.5 mM deoxynucleoside triphosphates (Invitrogen Life Technologies) and 200 units of reverse transcriptase enzyme (SuperScript RT II; Invitrogen Life Technologies). The mRNA expression levels were estimated using RT-qPCR (7500 PCR system; Applied Biosystems, Carlsbad, CA, USA) using specific primers for each molecule as follows (forward and reverse, respectively): TGF-β1 (5′-GCTGTGCAGGTGTTGAGCC-3′ and 5′-TCAGTCCCAAACGTCGAGGT-3′), interleukin (IL)-6 (5′-TGTATGAACAGCGATGATGCAC-3′ and 5′-GGTTATATCCAGTTTGGAAGCATCC-3′), IL-10 (5′-ATTGAACCACCCGGCATCTAC-3′ and 5′-GGTTTTCCAAGGAGTTGCTCC-3′). The relative expression of the target genes was normalized to the housekeeping gene β-actin (5′-CCTCTATGCCAACACAGTGC-3′ and 5′-ACATCTGCTGGAAGGTGGAC-3′). All primers were synthesized by Integrated DNA Technologies (Coralville, IA, USA). PCR product accumulation was monitored using SYBR Green I intercalating dye (Applied Biosystems, Warrington, UK), which exhibits increased fluorescence upon binding with double-stranded DNA.

### Western blot analysis

The kidney fragments were homogenized using a Polytron homogenizer (Kinematica, Lucerne, Switzerland) in ice-cold buffer [50 mM TRIS (Sigma-Aldrich), 150 mM NaCl (Labsynth), 1.0% nonidet-P-40 (Bio-Rad Laboratories, Inc., Hercules, CA, USA), 0.5% sodium deoxycholate (Sigma-Aldrich), 0.1% SDS, (pH 8.0; Sigma-Aldrich)] containing protease inhibitors (AEBSF, aprotinin, bestatin, E-64, leupeptin, pepstatin A) (Protease Inhibitor Cocktail; Sigma-Aldrich). Total protein was quantified using a modified Lowry method (Bio-Rad DC protein assay reagent; Bio-Rad Laboratories, Inc.). Protein samples (50 *μ*g) were separated according to size by 12% SDS-PAGE and electroblotted onto nitrocellulose membranes (GE Healthcare Life Sciences, Little Chalfont, UK). The membrane blots were probed with primary antibodies overnight at 4°C and with horseradish peroxidase (HRP)-conjugated secondary antibodies for 1 h at 4°C. The primary antibodies were obtained from the following sources: mouse monoclonal anti-GAPDH (cat. no. AM4300; 1:4,000; Ambion, Austin, TX, USA), mouse monoclonal anti-TGF-β1 (cat. no. T0438; 1:500; Sigma-Aldrich), mouse polyclonal anti-p47 phox (cat. no. 07–500; 1:1,000; Millipore, Billerica, MA, USA), rabbit polyclonal anti-CuZn superoxide dismutase (SOD) (cat. no. 07–403; 1:1,000; Millipore), rabbit polyclonal anti-renin (cat. no. sc-22752; 1:200; Santa Cruz Biotechnology, Inc., Santa Cruz, CA, USA), rabbit monoclonal anti-Smad3p (cat. no. ab52903; 1:500; Abcam, Cambridge, UK) and rabbit polyclonal anti-p65 NF-κB (cat. no. 06–418; 1:1,000; Millipore). The goat anti-rabbit (cat. no. NA934V; 1:20,000) and rabbit anti-mouse (cat. no. A9044; 1:60,000) HRP-conjugated secondary antibodies were purchased from GE Healthcare Life Sciences and Sigma-Aldrich, respectively. The protein bands were visualized using the Immobilon Western HRP substrate (Millipore). The obtained bands were quantified using the Luminescent Image Analyzer-LAS 4000 and Image Gauge V3.1 software (Fuji Photo Film Co, Tokyo, Japan).

### Light microscopy studies

The paraffin-embedded fragments were cut into 5-*μ*m sections using a rotary microtome (Leica, Herlev, Denmark). The tissue slides were deparaffinized in xylene three times (5 min each), and gradually rehydrated through a series of graded ethanol (100% twice for 5 min, 95% for 5 min, 70% for 5 min and 50% for 5 min). Histological sections were stained using picrosirius red staining kit (1% Sirius red in saturated picric acid; EasyPath, Indaiatuba, Brazil) for 24 h, or hematoxylin and eosin (Labsynth) and examined under light microscopy (Nikon Eclipse 2000 equipped with Nikon DS-Fi2; Nikon Corporation, Tokyo, Japan). The fibrotic area stained with picrosirius solution was quantified using Corel Photo-Paint 12 (CorelDRAW version 12; Corel Corporation, Ottawa, ON, Canada) and UTHSCSA - ImageTool software (version 3.0; University of Texas Health Science Center, San Antonio, TX, USA).

### Immunohistochemistry

The kidney slices were deparaffinized and rehydrated, as described above. To expose the antigens, the kidney sections were boiled in a target retrieval solution [citrate buffer (pH 6.0) for fibroblast-specific protein 1 (FSP1), TRIS buffer (pH 9.0) for α-smooth muscle actin (α-SMA) and ED-1] for 30 min. Endogenous peroxidase activity was blocked with 3% H_2_O_2_ (Labsynth) for 10 min at room temperature. Nonspecific binding was prevented by incubating the sections with a protein blocker (Dako, Carpinteria, CA, USA). The sections were incubated overnight at 4°C with primary antibodies: α-SMA (cat. no. A2547; 1:500; Sigma-Aldrich), FSP1 (cat. no. A5114; 1:400; Dako) or CD68/ED-1 (cat. no. MCA341R; 1:100; Serotec, Oxford, UK). Following washing with Tris-buffered saline (TBS) [50 mM TRIS, 150 mM NaCl], the sections were incubated with a horseradish peroxidase-conjugated polymer (Dako) for 30 min at room temperature. The slides were rinsed with TBS and the sites of antibody-antigen binding were visualized with 3,3′-diaminobenzidine (Dako). The sections were lightly counterstained with hematoxylin. The analyses were performed using light microscopy (Eclipse 2000 camera Nikon DS-Fi2) and the stained proteins were quantified using Corel Photo-Paint 12 (CorelDRAW version 12) and UTHSCSA-ImageTool software (version 3.0).

### Statistical analysis

Results are expressed as the mean ± standard error. The data were analyzed by SigmaStat 2.0 software (Systat Software Inc., San Jose, CA, USA), using one-way analysis of variance followed by Tukey’s test. P<0.05 was considered to indicate a statistically significant difference.

## Results

### Effect of calcitriol on proteinuria and serum markers

**Figure 1:**
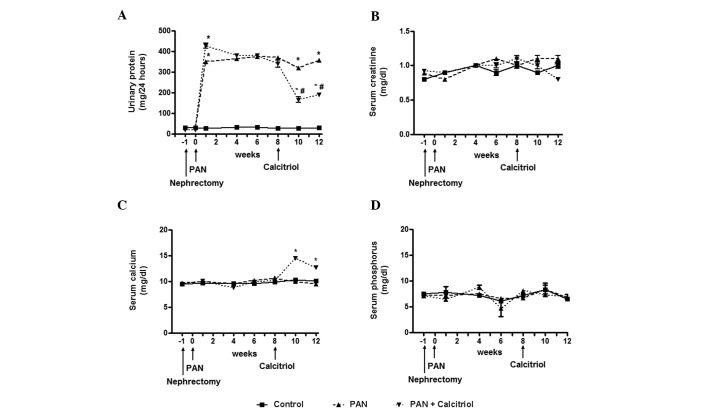
Effects of PAN and calcitriol on (A) urinary protein excretion (B) serum creatinine, (C) ionized serum calcium and (D) inorganic phosphate levels. The data are expressed as the mean ± standard error of the mean. Groups: Control (n=5), PAN (n=5), PAN + calcitriol (n=5). ^*^P<0.05 vs. control, ^#^P<0.05 vs. PAN. PAN, puromycin aminonucleoside.

As expected, one week after puromycin injection, intense proteinuria developed, which was significantly reduced by calcitriol treatment (Fig. 1A). Despite massive proteinuria, a change in the plasma creatinine concentration was not detectable (Fig. 1B). Treatment with calcitriol increased the serum calcium concentration at 10 weeks (Fig. 1C), which subsequently decreased at 12 weeks. There was no significant change in plasma phosphorus concentration (Fig. 1D).

### Calcitriol ameliorates renal damage and interstitial fibrosis

**Figure 2:**
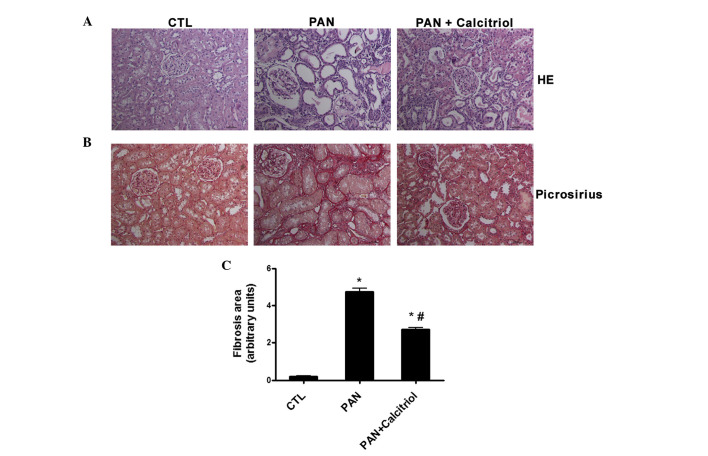
(A) Histological analysis of kidney sections stained with HE. (B) Histological analysis of kidney sections stained with picrosirius red, showing collagen deposition. Magnification, ×200. (C) Graphical representation of collagen deposition shown in B. The data are expressed as the mean ± standard error of the mean (n=5, for each group). ^*^P<0.05 vs. CTL, ^#^P<0.05 vs. PAN. PAN, puromycin aminonucleoside; HE, hematoxylin and eosin; CTL, control.

Kidney histology using H&E staining (Fig. 2A) revealed severe renal damage 12 weeks after puromycin administration, char-acterized by interstitial expansion and an increase in tubular lumen, possibly due to impaired tubular reabsorption. These alterations were minimized by calcitriol treatment (Fig. 2A). In addition, the weak collagen deposition detected in the tubulointerstitium and glomeruli in the nephrectomized control rats was markedly increased in the kidneys of the PAN-treated animals (Fig. 2B). Calcitriol administration was associated with significantly less collagen staining compared with that in the untreated proteinuric animals based on quantification of the picrosirius-positive areas (collagen deposition; Fig. 2C).

### Calcitriol attenuates fibroblast activation

**Figure 3:**
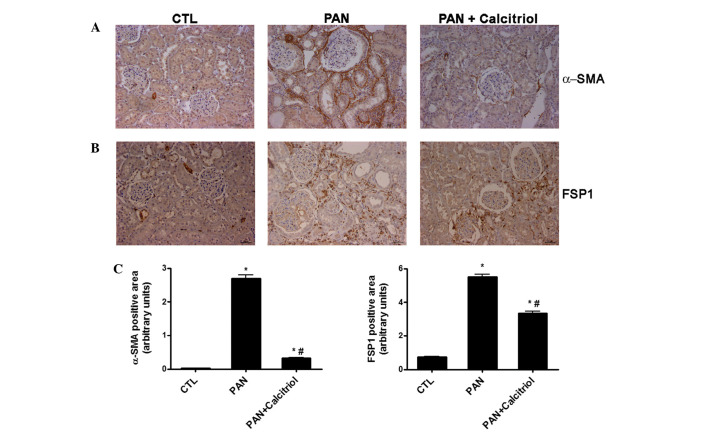
Representative microphotographs (magnification, ×200) showing cortical sections of the kidneys from the control, PAN and PAN + calcitriol groups. The tissues were probed with primary antibodies against (A) α-SMA and (B) FSP1. Magnification, ×200. (C) Quantitative analysis of the stained areas shown in panels A and B. Values are expressed as the mean ± standard error of the mean (n=5, for each group). ^*^P<0.05 vs. CTL, ^#^P<0.05 vs. PAN. PAN, puromycin aminonucleoside; CTL, control; FSP1, fibroblast specific protein 1; α-SMA, α-smooth muscle actin.

The expression levels of the fibroblast markers α-SMA and FSP1 were examined. There was an increase in the expression of the two fibroblast markers in the proteinuric animals (Fig. 3A and B). However, α-SMA was detected in the periglomerular region and interstitial space, while FSP1 staining was identified mainly in the interstitium. Calcitriol treatment reduced α-SMA and FSP1 expression, as shown in the semiquantitative analysis (Fig. 3C).

### Renoprotective mechanisms of calcitriol

**Figure 4:**
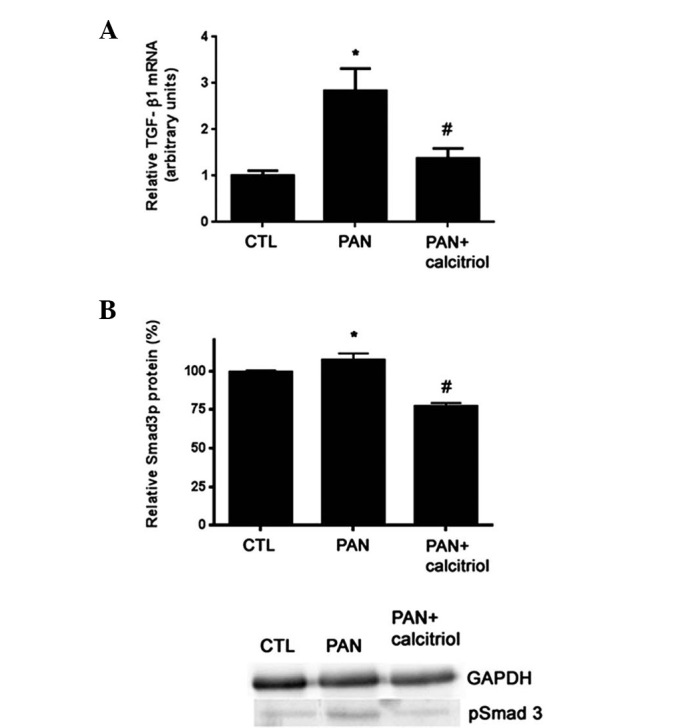
(A) TGF-β1 mRNA expression. (B) Western blot analysis of phosphorylated Smad3 protein expression. Values are expressed as the mean ± standard error of the mean (n=5, for each group). ^*^P<0.05 vs. CTL, ^#^P<0.05 vs. PAN. PAN, puromycin aminonucleoside; CTL, control; TGF, transforming growth factor.

**Figure 5:**
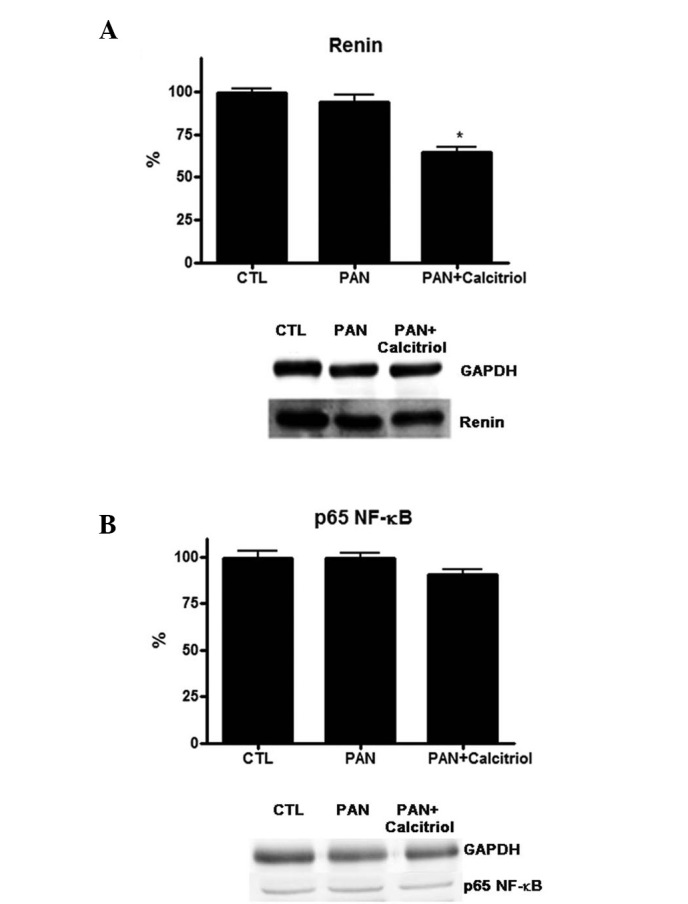
Representative western blots and quantitative determination of (A) renin protein and (B) NF-κB p65 protein expression. Membranes shown in B are the same as those used in Fig. 4B, which were stripped and reprobed with antibody against the NF-κB p65 subunit. Thus, the GAPDH control blot was the same as that used in Fig 4B. Values are expressed as the mean ± standard error of the mean (n=5, for each group). ^*^P<0.05 vs. CTL, ^#^P<0.05 vs. PAN. PAN, puromycin aminonucleoside; CTL, control; NF, nuclear factor.

The levels of TGF-β1 and the signaling molecule Smad3 were analyzed using RT-PCR and western blotting, respectively. Compared with the control rats, the proteinuric animals exhibited increased TGF-β1 mRNA expression and phosphorylated Smad3 (pSmad3) (Fig. 4). Calcitriol treatment significantly reduced TGF-β1 and pSmad3 expression. Regarding the function of RAS in this model, it was observed that PAN did not alter renin expression, but calcitriol treatment significantly reduced renin levels (Fig. 5A). Calcitriol was able to regulate the NF-κB pathway; however, no detectable alterations were observed for the NF-κB signaling protein p65 in the PAN and calcitriol groups (Fig. 5B).

### Calcitriol reduces renal inflammation

**Figure 6:**
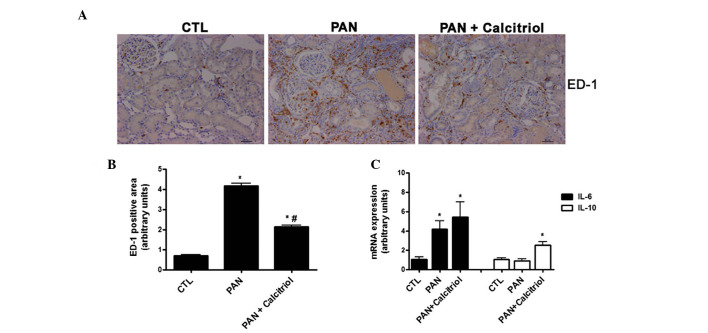
(A and B) Immunohistochemical staining and quantification of ED-1^+^ monocytes/macrophages in kidney sections. Magnification, ×200. (C) Gene expression of inflammatory cytokines (IL-6 and IL-10). The data are expressed as the mean ± standard error of the mean (n=5, for each group). ^*^P<0.05 vs. control, ^#^P<0.05 vs. PAN. PAN, puromycin aminonucleoside; CTL, control; IL, interleukin.

There was increased macrophage infiltration in the kidneys in the PAN group based on increased ED-1 staining (Fig. 6A and B). Calcitriol reduced the presence of macrophages, indicating a possible decrease in inflammation. There was increased gene expression of the proinflammatory cytokine IL-6 in the PAN group (Fig. 6C). Although calcitriol treatment did not change IL-6 expression, calcitriol significantly increased expression of the anti-inflammatory cytokine IL-10.

### Effect of calcitriol on oxidative stress

**Figure 7:**
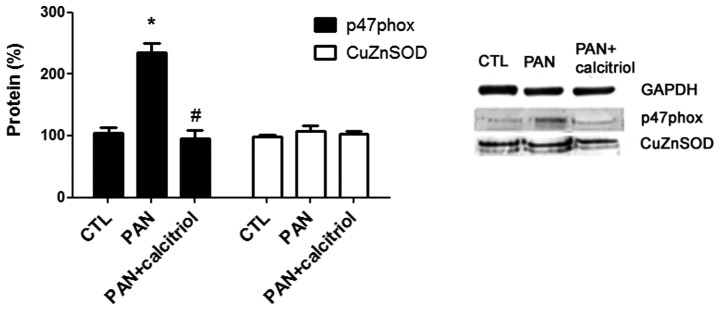
Western blot analyses showing the expression of two enzymes during oxidative stress. Quantitative determination and representative western blots for the oxidant enzyme p47 and antioxidant enzyme CuZnSOD. The data are expressed as the mean ± standard error of the mean (n=5, for each group). ^*^P<0.05 vs. control, ^#^P<0.05 vs. PAN. PAN, puromycin aminonucleoside; CTL, control; SOD, superoxide dismutase.

The mechanism of oxidative stress in the pathophysiology of puromycin nephropathy was assessed through analyzing the expression of two enzymes involved in this mechanism, p47 phox, a subunit of nicotinamide adenine dinucleotide phosphate (NADPH) oxidase and CuZnSOD, an antioxidant enzyme. There was a significant increase in p47 phox expression in the PAN group and calcitriol-treatment reduced this increase to near the control group level (Fig. 7). No significant differences in CuZnSOD expression were identified.

## Discussion

PAN-induced nephropathy is characterized by podocyte injury, resulting in glomerulosclerosis, tubular damage and interstitial fibrosis. PAN nephropathy was reproduced in a rat model in the present study and was characterized by nephrotic level proteinuria with no detectable change in serum creatinine. Calcitriol administered eight weeks after PAN-induced renal injury significantly reduced proteinuria. Although podocyte function and morphology was not evaluated in the present study, a previous study demonstrated that the vitamin D analogue paricalcitol may prevent podocyte lesions in adriamycin nephropathy (5) and the reno-protective effect of paricalcitol was considered to be primarily due to the prevention of podocyte injury.

The protein overload in the tubules resulted in epithelial cell damage with functional and structural changes, including EMT (11). In the present study, there was a significant change in tubular structure with lumen dilation, indicating impaired reabsorptive capacity of the tubular epithelial cells. Although PAN induced an increase in the EMT markers FSP1 and α-SMA, these markers were predominately identified in interstitial cells but not in tubular cells. This finding suggested that EMT was not the main mechanism of fibrosis in this model, although the involvement of EMT in interstitial fibrosis cannot be fully ruled out. Calcitriol treatment was able to minimize the overexpression of FSP1 and α-SMA induced by PAN, suggesting that the beneficial effects of calcitriol supplementation on fibrogenesis were mediated, at least in part, by reduced fibroblast activation, although the origin of fibroblasts, either resident and/or infiltrating, was not determined in the present study.

Calcitriol was able to improve renal morphology and reduce the fibrotic area with less collagen deposition. TGF-β1, one of the most relevant profibrotic factors in the kidney, was increased by PAN as well as its signaling pathway, represented by pSmad3, which was activated by puromycin. Calcitriol completely inhibited this pro-fibrotic mechanism, suggesting that the actions of vitamin D in renal fibrosis may involve a downregulation of the TGF-β1/Smad3 axis.

Chronic inflammation is an important mechanism in tissue injury and fibrogenesis (12). PAN induced increased macrophage infiltration with increased expression of the pro-inflammatory cytokine IL-6. Calcitriol was shown to have a potent anti-inflammatory effect in different experimental models of kidney disease (13,14). Additionally, clinical studies have demonstrated that calcitriol treatment suppressed IL-6 and TNF-α expression in patients with chronic kidney disease (15). In the present study, calcitriol reduced the presence of ED-1-positive cells, indicating less macrophage infiltration; however, calcitriol did not reduce IL-6 mRNA expression, but of note, calcitriol upregulated the anti-inflammatory cytokine IL-10, suggesting an indirect effect of calcitriol in PAN-induced inflammation.

Reactive oxygen species formed by oxidative stress are important mediators of renal disease induced by puro-mycin (16). The results of the present study demonstrated the presence of PAN-induced oxidative stress represented by a significant increase in the expression of p47phox, an NADPH oxidase subunit, with no change in SOD expression, resulting in an imbalance between prooxidant and antioxidant mechanisms. Treatment with calcitriol decreased p47phox expression and thus reduced oxidative stress. Finch *et al* (17) reported that paricalcitol ameliorated oxidative stress by increasing CuZnSOD expression in uremic rats. In addition, treatment with an antioxidant attenuated renal interstitial fibrosis following ureteral obstruction (18), indicating a function for the redox state in fibrosis progression. In the present study, the effect of calcitriol in reducing oxidative stress was mediated, at least in part, via TGF-β downregulation.

There is evidence of puromycin-induced RAS activation in nephropathy (19); however, intrarenal renin expression did not change in the present model. Calcitriol has been well accepted to suppress prorenin gene expression and reduce RAS activity and although PAN does not have an effect on renin expression, calcitriol induced a 30% decrease in renin protein expression, but the impact of this reduction on the renoprotective effect of calcitriol in the present study remains elusive.

In conclusion, although the functional and histological parameters did not completely return to control levels, calcitriol treatment significantly ameliorated the progression of puromycin-induced renal fibrosis. In addition, calcitriol was effective in decreasing the accumulation of extracellular matrix, reduced inflammation and downregulated the TGF-β1 pathway. Therefore, calcitriol supplementation may be a strategy to reduce renal damage in proteinuric kidney disease.
